# Baseline Human Metabolic Profiling and Risk of Death from COVID-19: Conceptualization of Multivariate Prediction Model Development via Retrospective Database Analysis in the United States Department of Veterans Affairs

**DOI:** 10.3390/jcm15031212

**Published:** 2026-02-04

**Authors:** Heather M. Campbell, Allison E. Murata, Jenny T. Mao, Benjamin McMahon, Glen H. Murata

**Affiliations:** 1 VA Cooperative Studies Program Clinical Research Pharmacy Coordinating Center, 2401 Centre Avenue SE, Albuquerque, NM 87106, USA; allison.murata@va.gov; 2 College of Pharmacy, University of New Mexico, MSC09 5360, 1 University of New Mexico, Albuquerque, NM 87131, USA; 3 New Mexico VA Health Care System, 1501 San Pedro Dr SE, Albuquerque, NM 87108, USA; jenny.mao@va.gov (J.T.M.); ghmurata@aol.com (G.H.M.); 4 Division of Pulmonary, Critical Care and Sleep Medicine, School of Medicine, University of New Mexico, 2211 Lomas Blvd NE, Albuquerque, NM 87106, USA; 5 VA San Diego Healthcare System, 3350 La Jolla Village Dr., San Diego, CA 92161, USA; 6 Division of Pulmonary, Critical Care, Sleep Medicine and Physiology, School of Medicine, University of California San Diego, 9500 Gilman Drive, La Jolla, CA 92093, USA; 7 Los Alamos National Laboratory, Bikini Atoll Rd. SM 30, Los Alamos, NM 87545, USA; mcmahon@lanl.gov; 8 Independent Researcher, Highlands Ranch, CO 80130, USA

**Keywords:** risk adjustment, prognosis, clinical chemistry tests, vital signs, biomarkers, medical informatics, big data, veterans, COVID-19

## Abstract

**Background/Objectives**: Prediction models are implemented frequently, yet, compared with other study designs, their incorporation of clinical measurements (CMs; i.e., vital signs and laboratory results) is rather underdeveloped. The purpose is to describe methods used and illustrate clinical utility in parameters systematically derived from CMs; as a case study, we use the risk of all-cause mortality following coronavirus disease 2019 (COVID-19) as the basis for prognosis. **Methods**: We identified cases through the Department of Veterans Affairs COVID-19 Shared Data Resource, utilizing data from the first visit until 14 days before testing positive. Thirteen parameters were derived from each of the 11 CMs, capturing departures from normality considering variability and time. The 143 candidate predictors were used to generate the main logistic regression model. The area under the receiver operating characteristic curve (AUROC) analysis was performed to assess discrimination between those who lived and died for subset and main regressions; for comparison, this was performed for an age-only model and the Charlson Comorbidity and Elixhauser Indices. **Results**: There were 329,491 patients. The main model’s AUROC (0.785 ± 0.002) was similar to the age-only model (0.783 ± 0.002; *p* > 0.05) and significantly greater than the comorbidity indices’ (range: 0.675 ± 0.002 to 0.729 ± 0.002; *p* < 0.001 each). **Conclusions**: The study found several parameters were significant determinants of mortality following COVID-19, highlighting the importance of a systematic approach for multivariate modeling to obtain informative insights into underlying pathophysiology. The main model outperforms common comorbidity indices as a summary metric for pre-existing conditions in this case study. If validated, this approach could revolutionize the way CMs are handled in multivariate models.

## 1. Introduction

Prediction models are gaining traction because they can be a foundation for risk stratification, triaging patients according to who is most likely to receive benefit [1]. Additional insight could be gleaned if clinical measurements (CMs) were prominent in these models: CMs include vital signs and laboratory tests and can provide valuable information about the metabolic status of patients at baseline. Specifically, (a) a diagnosis informs that the patient has a certain disease while CMs reveal the burden imposed by its risk factors or the disease itself, (b) CMs provide insight into the mechanism of injury and (c) CMs add explanatory variables to a prediction model. Of the three, the last warrants more explanation; adding CMs to prediction models is critical to identifying targets for interventions to reduce the risk. For example, causality may be inferred by a dose–response relationship between the CM and risk of mortality. The model would become hypothesis-generating, possibly leading to other studies confirming the mechanism of injury or finding treatments that reduce the risk. The current state of prediction models reveals that they rarely define causality such that they point to further courses of action in this way. A review of the literature revealed that only two prediction models combined several CMs to predict mortality [2,3]. Both models incorporated CMs from one timepoint and provided risk scores.

Publications of studies other than prediction models have reported variables derived from CMs in addition to the most recent value, allowing incorporation of time and variability, revealing significant, independent associations. For instance, investigators of a systematic review [4] found significant associations in short-, medium- and long- term blood pressure variability with all-cause mortality after adjusting for mean blood pressure. Similarly, short- and long- term variability in glycosylated hemoglobin (A1c) has been significantly associated with all-cause mortality as well as micro- and macro- vascular complications [5,6,7,8,9]. The studies from these two CMs employed different measures of variability, including standard deviation, standardized residual, coefficient of variation, variation independent of the mean, percent of consecutive CMs that differed by a predetermined threshold, percent of time CMs were within target range, percent of time CMs were above range, percent of time CMs were below range and root mean squared error, acknowledging the importance of incorporating this data while simultaneously implying lack of consensus concerning appropriate variables to represent such data in the medical literature [4,10]. It is intuitive that capturing the departure from normal or reference values in terms of magnitude and time in different ways may lead to a better understanding of the underlying pathophysiology [11]. Studies assessing these variables have been piecemeal, looking at one to three of these variables [12,13,14,15,16,17,18,19,20,21,22,23]. Except for one study [18], which used data from two CMs, each assessed only one CM, providing an opportunity to incorporate variables into a more comprehensive model evaluating their potential relationship with prognosis. This is important in two ways. First, disease progression is based on many factors, acting through different mechanisms of injury. Second, more than one-quarter of adults in the United States (U.S.) had at least two chronic diseases in 2018; this is expected to double among those aged 50 years and older by 2050 [24]. With prediction models becoming more mainstream and their inclusion of this type of information being relatively underdeveloped, we sought to develop methods supporting this needed systematic approach.

**Figure 1:**
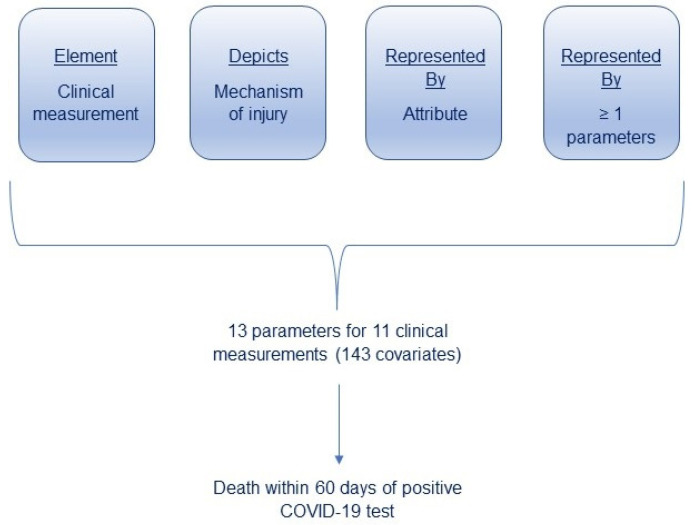
Conceptualization of Study Design. Clinical measurements are listed in the first paragraph of the Section 2. Mechanisms of injury are subsequently found within the paragraphs of each attribute. Each attribute is identified by bold italicized words (a) through (g). Each parameter can be identified as italicized terms within each attribute.

Our approach emulates the way clinicians interpret CMs to reach conclusions about patient metabolic status. Clinicians rarely interpret individual values for CMs without review of neighboring values or other domains of the medical record. Moreover, clinicians often render judgments about several attributes when assessing a CM. Each concept is independent of the others and has clinical implications of its own. For example, a patient with diabetes may have an A1c that meets a metabolic target, but the entire value set could represent a substantial glycemic burden, indicate a tendency to relapse and demonstrate a steep upward trend. In the current study, a parameter is a variable synthesized from the complete value set for a CM that represents one attribute for that CM. To reach conclusions about metabolic status requires extensive processing of all data for a CM, deriving parameters for each attribute and testing the association between derived parameters and the outcome of interest. Figure 1 conceptualizes the interplay between these components as they relate to the study design. The algorithms used in this study derive these parameters and generate a running summary whenever a new value is added to the set. Thus, the patient’s status relative to any CM can be assessed at any point in time. The purpose of this paper is to describe the methods used for summarizing the effects of values from each patient for each CM. As a case study, we illustrate the clinical utility of using the risk of death following coronavirus disease 2019 (COVID-19) as the basis for prognosis.

## 2. Materials and Methods

We followed Transparent Reporting of a multivariate prediction model for Individual Prognosis or Diagnosis (TRIPOD) recommendations for development. Cases were identified through VA’s COVID-19 Shared Data Resource (CSDR). Membership in this nationwide registry requires at least one positive nucleic acid amplification test in or external to the VA; the VA had 1731 sites across the U.S. in 2001. The primary outcome was all-cause mortality within 60 days of the first positive result. The outcome was retrieved from the CSDR, which assigns a 1 to those who died and 0 otherwise. Starting in 1997, data domains in the Corporate Data Warehouse (CDW) were interrogated for all measurements entered from the first visit in the VA until 14 days before the diagnosis of COVID-19. This precaution excludes readings that may have been taken during the pre-symptomatic phases of illness. Using CMs routinely collected in periodic health examinations, we retrieved systolic blood pressure (SBP), diastolic blood pressure (DBP), oxygen saturation (O_2_Sat), height and weight from the file Vital.VitalSign. The latter were used to calculate body mass index (BMI). We also retrieved estimated glomerular filtration rate (eGFR), albuminuria (ALB), hematocrit (HCT), alanine aminotransferase (ALT), A1c, high-density lipoprotein (HDL) and low-density lipoprotein (LDL) from the file Chem.PatientLabChem. The following parameters were derived for each of the 11 CMs (above), categorized by attribute:

**(a) *Current metabolic control*** requires that the measurement be compared to an external standard, that both be standardized to a common scale, that the value is timely, that it has not already been enacted upon, that enough time has elapsed since the last treatment change to reach a plateau value and that it is not rapidly increasing or decreasing. Current metabolic control, or lack thereof, indicating severity, is reflected in the most recent value (*Value1*). The programs used in this study provide the option of raw or standardized values and require the user to specify a treatment target.

With the former parameter being the most straightforward, we turn to more complicated parameters employed to reflect the remaining attributes. In doing so, we reference studies using parameters to establish a baseline with pre-existing long-term data, as appropriate; some require more context than others, as we were unable to identify prior use of some parameters. In this vein, although the existing literature includes time in the therapeutic range of SBP [12,13,14], frequency in the range of SBP [13], number of CM values at goal in ALT [15] and A1c [16] and time above and below range in A1c [10], we refine these parameters to discern between chronicity and refractoriness of disease, which in turn provides insight into other attributes, as described below.

**(b) *Chronicity*** is defined as the total number of days or measurements above or below the target, as appropriate. Each reading was paired with the preceding one. Days between successive abnormal readings were considered not at goal, days between normal readings as at goal, days between a normal and abnormal reading as worsening and days between an abnormal and normal reading as improving. Days not at goal were summarized across all successive pairs in the patient record (*FUDaysNotAtGoal*). *NumNotAtGoal* is the total number of measurements outside the target value in the patient record.

**(c) *Disease burden*** is the degree to which an abnormality has caused end-organ damage. For chronic diseases, it is almost always a function of severity and exposure time, expressed as the area under the severity x time curve (AUC). AUC has been used by Domanski et al. (2020) for assessing the relationship between LDL and incident CVD events [17]. The software employed in this study uses a simple trapezoidal estimation technique without interpolation, extrapolation or curve-fitting and includes all readings from the first to the last value. AUC can be large just because the patient is elderly. To eliminate this possibility, we derived AUC only outside the target specified by the user (*AbnAUC*), similar to the method by Liu et al. (2021), who used it for LDL and non-HDL for incident peripheral artery disease, and Li et al. (2022), who used it for BMI to assess colorectal cancer risk [18,19]. Time-weighted averaging has been used for evaluating the relationships of LDL with CVD events and mortality [20]. It eliminates the sampling bias that arises from a tendency to measure a CM more frequently when it is abnormal [25]. The raw average is an overestimate of severity when abnormal readings are more closely spaced than normal ones. *TimeWtAvg* is defined as follows: AUC/(Total days of follow-up).

**(d) *Refractoriness*** is the repeated failure to achieve a goal and is represented by consecutive abnormal values (a “cluster”). Each cluster begins with a change from a normal to an abnormal value and terminates with the next normal value. The assumption is that an abnormal reading should have triggered an intervention that normalized the measurement. Refractoriness is worse when there are many readings in the cluster, or its duration is long. It can result from the failure to treat, failure of the patient to adhere, failure of the disease to respond, or intolerable side effects. Our program defines a start date, consecutive days not at goal (*TimeNotAtGoal*) and consecutive readings not at goal (*CtNotAtGoal*) for each cluster. The least refractory episode has a *CtNotAtGoal* of one, and refractoriness increases as *CtNotAtGoal* increases.

**(e) *Tendency to relapse*** is based on the occurrence of abnormal values after being at goal. Relapses can occur one or many times and are often caused by a steady progression in disease severity, which requires multiple titrations of treatment. The number of relapses is represented by *NumClust*. The first abnormal cluster is given the value of one; patients with a tendency to relapse have larger values for *NumClust*. Relapse and refractoriness have different clinical implications. For example, a person can relapse many times, respond quickly to each intervention and spend little time not at goal. On the other hand, a person with refractoriness can have just one cluster that lasts for years.

**(f) *Lability*** refers to variations in a CM from one reading to the next. Spontaneous variation can occur when the test is imprecise or when there are fluctuations in the CM itself. Test imprecision may be due to changes in conditions of testing, often behavioral, or variability in the assay. Results of these tests should be confirmed by repeated sampling. On the other hand, physiologic variability results from a complex relationship among multiple control mechanisms and is important in several conditions. The coefficient of variation (*CoeffVar*) for each CM is derived from the grand mean (GrandMean) and standard deviation (GrandStD) for all values in the patient record and given by the expression GrandStD/GrandMean. Chia et al. (2016) use these parameters in the context of labile hypertension, finding significant associations with it and progressive renal dysfunction [21]. *MeanValDiff* refers to the average of the absolute difference between all consecutive pairs.

**(g) *Temporal trends*** refer to changes over time from one value to another and may be long- or short-term. *NetChange* is a measure of long-term trends and refers to the difference between the most recent and first value for each CM, approached similarly to Nakazawa et al. (2022) for eGFR for renal dysfunction [22]. The software generates the mean of the 3 lagging values for each measurement (Lag3Mean). *Lag3Dev* refers to the fractional deviation of the current value from Lag3Mean and is given by the following expression: (Value1—Lag3Mean)/Lag3Mean. One can think of these two parameters as akin to using data for a moving average to reveal longer-term patterns than the change in two sequential CMs and reveal shorter-term patterns of clinical significance than the overall history, by comparing it to the most recent value.

To review, we analyzed the following 13 parameters for each CM: *Value1*, *FUDaysNotAtGoal*, *NumNotAtGoal*, *AbnAUC*, *TimeWtAvg*, *NumClust*, *TimeNotAtGoal*, *CtNotAtGoal*, *MaxClustDays*, *CoeffVar*, *MeanValDiff*, *NetChange* and *Lag3Dev*. These values were extracted for each patient from the running summary associated with the most recent value. The default was to use all available readings in the medical record so that metabolic status was defined over the patient’s lifetime. Computer code, though not available, was validated. Table 1 depicts a summary of parameters by attribute.

### Statistical Methods

Since there are 11 CMs of interest, the list of candidate predictors consists of 13×11=143 items. Group differences in categorical variables were tested by chi-square analysis. Group differences in continuous variables were analyzed by Student’s *t*-test and Mann–Whitney U-test.

For each CM, a subset logistic regression was performed to identify which of the 13 parameters were predictive of mortality. A variable was considered significant if the adjusted *p*-value associated with the coefficient was <0.05. The subset model was then used to assign a predicted probability of death to each patient. Area under the receiver operating characteristic curve (AUROC) analysis was conducted to determine if the predicted probability discriminated between those who lived and died.

Then, the 143 candidate predictors were used to generate a main predictive model. Stepwise logistic regression was applied. Modeling started with the set of the most recent values. Sets related to different criteria were added in succession; if variables that previously entered the model became insignificant, they were removed. A variable was considered significant if the adjusted *p*-value associated with its coefficient was <0.05. The final model was used to assign a predicted probability of death (PDeathLabs) to each patient based upon all CMs. Again, AUROC analysis was used to determine if PDeathLabs was able to identify those who died. For comparison, single-factor logistic models were developed for age at diagnosis, the 2-year Charlson Comorbidity Index score (Charl2Yrs), lifetime Charlson score (CharlEver), the Elixhauser 2-year score (Elix2Yrs) and lifetime Elixhauser score (ElixEver). Their predicted probabilities were used to derive an AUROC for each.

Using CMs routinely obtained in periodic health examinations over a long time of collection, a few missing values for CMs were anticipated. With the primary outcome being mortality and the knowledge of at least some of the CMs being risk factors for death, specifically as expressed through *Value1*, patients of interest were those who had at least one abnormal CM at the time of testing positive. Furthermore, chronic disease management was of interest. Therefore, case-wise deletion was used for subset regressions and the main predictive model, making *TimeNotAtGoal* and *CtNotAtGoal* relevant only for patients with an abnormal CM at the time of testing positive. (Patients with a normal CM at that time would have missing values.) Similarly, patients who did not have at least 2 consecutive abnormal values for a CM would have missing values for *MaxClustDays.* Accordingly, study findings pertain to patients with an abnormal CM at the time of testing positive who had at least 2 consecutive abnormal values for a CM at any point during CM collection.

Code was developed and validated in SQL Server Management Studio 18 (Microsoft, Redmond, WA, USA). All statistical analysis was conducted in StataMP 17 (StataCorp LLC, College Station, TX, USA).

## 3. Results

On 30 September 2021, there were 347,220 COVID-19 patients in VA’s CSDR. Of these, 329,491 patients (94.9%) had CMs at least 14 days prior to testing positive. The mean age at the time of diagnosis was 59.1 ± 16.6 years, 85.5% were male, 23.4% were members of a racial minority, 9.2% were Hispanic, 96.4% were veterans, 0.7% were on supplemental oxygen, 12.2% were current smokers and 9.3% had been fully vaccinated at least 14 days prior. Of those meeting eligibility criteria, 21.6% acquired their infections after 1 July 2021. Overall, 17,934 patients (5.44%) died within 60 days of their diagnosis.

Among the subset regressions performed, all models but the one for ALT converged (Table 2, Table 3, Table 4, Table 5, Table 6, Table 7, Table 8, Table 9, Table 10 and Table 11). Some AUROCs were close to those of comorbidity indices (i.e., DBP: 0.705 ± 0.002, eGFR: 0.691 ± 0.002, albuminuria: 0.693 ± 0.002, LDL: 0.675 ± 0.002, comorbidity indices’ range: 0.675 ± 0.002 to 0.729 ± 0.002). The number of significant parameters ranged from 7 for HDL to 13 for SBP. *Value1* was significant for 7 CMs, *FUDaysNotAtGoal* for 9, *NumNotAtGoal* for 9, *AbnAUC* for 9, *TimeWtAvg* for 9, *NumClust* for 10, *TimeNotAtGoal* for 6, *CtNotAtGoal* for 7, *MaxClustDays* for 5, *CoeffVar* for 10, *MeanValDiff* for 10, *NetChange* for 9 and *Lag3Dev* for 7.

In the cohort, 239,393 patients had complete sets of data for developing the main model (27.3% missing). Table 12 shows the components of this model. Of 143 candidate predictors, 49 parameters were identified as statistically significant, independent predictors of all-cause mortality. The main model was used to calculate a predicted probability of death (PDeathLabs) for each subject. The AUROC for PDeathLabs was 0.785 ± 0.002. No difference was found between the AUROC of PDeathLabs and age at diagnosis (0.783 ± 0.002; *p* > 0.05). However, the AUROC for PDeathLabs was significantly greater than that of Charl2Yrs (0.704 ± 0.002; *p* < 0.001), CharlEver (0.729 ± 0.002; *p* < 0.001), Elix2Yrs (0.675 ± 0.002; *p* < 0.001) and ElixEver (0.707 ± 0.002; *p* < 0.001). Thus, baseline metabolic measurements outperform common comorbidity indices for pre-existing conditions in predicting mortality following COVID-19.

## 4. Discussion

We propose a comprehensive method for handling pre-existing findings from vital signs and laboratory tests in models predicting mortality following COVID-19. We believe the parameters *MaxClustDays* and *NumClust* are novel in that we were unable to identify any studies using them or describing their development. Their prevalent significance across CMs in the subset and main models provides evidence of their value and encouragement for future use: in the main model, 7 of 49 (14.3%) parameters constituted these. In line with Maziarz et al. (2017), our observations suggest a variety of parameters derived from CMs capturing departure from normal or reference values in terms of magnitude and time should be included because they have independent prognostic significance, generate insights into the mechanism of action and may be targets for interventions to mitigate the risk [11]. By including explanatory variables, models can become hypothesis-generating and lead to future studies validating the suspected pathogenesis or interventions targeting the abnormality itself.

The 13 parameters were identified as predictors of mortality in the subset models, revealing convergence between the approach used in clinical practice and CM parameters used in this study. Our main model showed that many parameters besides the most recent value were significant contributors to mortality following COVID-19. For 2 CMs (i.e., LDL and eGFR), disease burden (*AbnAUC* and *TimeWtAvg*) contributed to the prediction while the most recent value (*Value1*) did not. For 6 others (i.e., SBP, BMI, HDL, O_2_Sat, DBP and ALT), both current metabolic control (*Value1*) and long-term parameters (*NumNotAtGoal*, *FUDaysNotAtGoal*, *AbnAUC*, *TimeWtAvg* and *NetChange*) were independently predictive of death. This finding suggests that CMs have chronic and acute effects on mortality following COVID-19 and that the former should be routinely considered for prediction models. Our results confirm the importance of CM parameters for COVID-19 prognosis, validate the attributes they represent and underscore the hazards of solely using one parameter or using parameters irrespective of time.

The complex interplay of different body systems is revealed by comparing the subset regressions to the main model. When solely assessing parameters relating to one CM, the most influential attributes were lability, disease burden, chronicity and temporal trends. However, when assessing all CMs routinely collected in these patients, the most influential attributes were current metabolic control, disease burden, temporal trends and tendency to relapse, providing further evidence of the importance of assessing both attributes and CMs more systematically. Higher values for O_2_SatValue1 and O_2_SatTimeWtAvg in our main model independently decreased the risk of mortality. This observation implies that the metabolic consequences of acute hypoxia and the burden imposed by chronic hypoxia create a substrate for COVID-19 injury. The latter may have done so through secondary pulmonary hypertension or may have been a marker for severe lung disease. When looking at the main model of the BP measures of hypertension severity, only SBPTimeWtAvg was a risk factor for mortality following COVID-19. Higher values for DBPValue1 and DBPTimeWtAvg were protective. This paradoxical effect might be explained by the fact that, for a given level of risk posed by SBP, a higher DBP is associated with a lower pulse pressure, reduced systolic wall stress and better coronary perfusion. This analysis suggests that the most important mechanism is long-term elevation of SBP. In a study systematically assessing all documented pre-existing conditions, hypertension was the most important independent risk factor for mortality following COVID-19 [26].

BMI and obesity are considered risk factors for mortality following COVID-19 [27,28]. However, we found that higher values for BMIValue1 in the main model lowered the risk of death. This discrepancy might be explained by the fact that we controlled for the mediators of injury in obesity (SBP, DBP, A1c, HDL and LDL). Once these factors are considered, a higher BMI may only be a marker of better nutrition. This finding illustrates the hazards of drawing conclusions from incompletely specified models. Serum albumin and hematocrit are indicators of nutritional status or catabolism and are powerful predictors of recovery from acute illness. On the other hand, they do not produce cumulative organ injury over the years. Accordingly, AlbValue1 and HCTValue1 were included in the main model with only one of their long-term parameters. A high value for A1cValue1 was identified as a risk factor, while measures of disease burden were not. Thus, recent hyperglycemia is deleterious in COVID-19 infection but microvascular injury developing over decades may not play that much of a role. Likewise, HDLValue1 had a protective effect while the long-term parameters did not. Finally, we found that time-weighted average and abnormal AUC were both included as measures of disease burden for eGFR, ALT, LDL and O_2_Sat. These results imply that abnormal AUC provides information about risk independent of the time-weighted average. This study shows there is no justification for arbitrarily selecting one parameter of a CM for modeling unless there is a physiologic basis for doing so, even when more than one parameter describes the same clinical attribute.

We also found that temporal instability in a CM, whether reflected in long- or short-term trends, coefficient of variation, or tendency to relapse, had significant effects on prognosis. The direction and magnitude of this effect varied across CMs and, at times, were counterintuitive. Although counterintuitive findings may indicate true effect, they may also suggest the presence of residual confounding. Specific to COVID-19, future studies should be conducted to identify the mechanisms by which fluctuations in CMs affect mortality. Until then, it is reasonable to include temporal changes in CMs in such models. This recommendation is consistent with the way that clinicians prioritize treatment of abnormal values. For example, many would consider a patient whose A1c rapidly increased from 5.0% to 8.5% to have a poorer prognosis than one whose A1c decreased from 11.0% to 9.0%.

As expected, parameters of chronicity (*FUDaysNotAtGoal* and *NumNotAtGoal*) added little to the model once disease burden was considered, with 1 and 3 terms entering the main model, respectively. Refractoriness was shown to have minimal contribution to death as well. First, without any terms entering the model for *MaxClustDays*, the length of the longest duration of abnormal values for any CM had no impact on death. Second, the number of consecutive abnormal values and the length of time of abnormal values had minimal effect on death. This is evidenced by 1 and 2 terms entering the main model for *CtNotAtGoal* and *TimeNotAtGoal*, respectively. Nevertheless, refractoriness remains a useful clinical concept because it could matter for chronic disease management. The situation with refractoriness is juxtaposed with the tendency to relapse; the latter attribute had 7 terms (*NumClust*) appear in the main model, underscoring the value of clustering and being another attribute revealing the importance of the length of time collecting CMs.

We calculated a predicted probability of death (PDeathLabs) based on complete value sets for the 11 CMs. The AUROC indicated that vital signs and laboratory findings are powerful predictors of outcomes. In fact, the AUROC for PDeathLabs and age at diagnosis were equivalent, while PDeathLabs was consistently superior to Charl2Yrs, CharlEver, Elix2Yrs and ElixEver. With the Charlson Comorbidity Index being noted as the second-most frequently used risk stratification tool in a recent review [1], these comparisons reveal the potential for PDeathLabs in clinical practice, should it perform as well in other health conditions and populations. In summary, the term generated with our approach is a convenient way to summarize millions of observations for CMs performed on hundreds of thousands of patients. Its greatest utility would be as a covariate in a parent model containing other domains. Unlike conventional comorbidity indices and the only prediction models identified that incorporate several CMs to predict mortality, PDeathLabs provides patient-specific probability of outcome occurrence.

Collectively, the interpretation of parameters and CMs with respect to mortality following COVID-19, placed in the context of two frequently used measures of risk, shows the promise of using such a systematic approach, not only in terms of the number of parameters representing several attributes, but also the number of CMs representing several physiologic mechanisms. The parameters in this study included those previously used in studies as well as novel parameters. We build upon the work by Doumas et al. (2017); they assessed the percentage of blood pressure readings in the therapeutic range over ten years for an association with mortality in about 700,000 patients at fifteen VA Medical Centers, finding a significant relationship [23]. By also looking at several timepoints of CM values, we expanded the number of parameters related to the inverse of values in therapeutic range to assess chronicity, disease burden, refractoriness, relapses, lability and temporal trends. To this end, we provide unique contributions in comparison to Chen et al. (2024); they looked at the number of alanine aminotransferase (ALT) values over a certain threshold, finding a significant positive relationship between it and the incidence of metabolic-dysfunction-associated fatty liver disease (MAFLD) in multivariate regression analysis [15]. Whereas they evaluated annual data over three years of ALT values before following for MAFLD, by virtue of our cohort having more CMs over a longer period, we were able to refine such out-of-range values in relation to each cluster for each CM to assess refractoriness. The expansion of parameters used in this study is advocated by two groups of investigators who measured similar phenomena. Kodani et al. (2023) noted that BP variability was more strongly related to adverse events than BP consistency [13]. Xu et al. (2023) provided a rationale for their measure compared to more typically reported ones by expressing the need to control for sicker patient bias; in the current study, this was handled through time-weighted averages [16].

We did not include age in our model for PDeathLabs for several reasons. First, we believed it was imperative not to have age supplant those clinical parameters highly correlated with age, such as blood pressure or weight [26]. Retaining parameters in the model allows for more insight into the physiologic mechanisms than a proxy for disease, such as age. To understand the mechanisms that lead to a fatal outcome, explanatory variables should take precedence over disease markers. They may even be targets for an intervention. Relatedly, models based on demographics do not support decision-making at the point of care. For example, if an elderly patient presents with refractory hypoxemia or terminal renal failure, a physician would propose withdrawing care based on dismal prognosis rather than age, if necessary. Clinicians make recommendations based upon assessment of the underlying conditions. It is the patient’s prerogative to decide what is appropriate based upon age and quality of life.

### Limitations

Our conclusions are limited to patients with characteristics similar to the veteran population, who are older and predominantly male. Having said this, similarities exist between the veteran and Medicare populations [29]. Moreover, higher PDeathLabs identifies those who tend to have chronic illness through two mechanisms. First, many parameters found to be significant required multiple measurements. Second, those followed for longer periods of time have higher values for the parameter even if the absolute elevations are the same. It is unclear if our conclusions are applicable to patients without such indications. Further studies should be conducted on other populations and disease states before the method is widely applied. If validated, our method could revolutionize the way in which CMs are handled in multivariate models.

## 5. Conclusions

Our study found that several parameters derived from 11 CMs were significant determinants of mortality following COVID-19, underscoring the importance of a systematic approach for multivariate modeling, emulating clinician thought to obtain more informative insights into the underlying pathophysiology. PDeathLabs has the same discriminating power as age at diagnosis and outperforms comorbidity indices as a summary metric for pre-existing conditions. If validated by others, this approach could provide a robust approach to handling CMs in multivariate models.

## Figures and Tables

**Table 1:** Summary of parameters by attribute.

Attribute	Parameter	Definition
Current metabolic control	Value1	Most recent value
Chronicity	Follow-up (FU)DaysNotAtGoal	Days not at goal across all successive pairs in the patient record
	Number (Num)NotAtGoal	Total number of measurements outside the target value in the patient record
Disease burden	Abnormal area under the curve (AbnAUC)	Area under the severity x time curve that is outside the specified target value
	TimeWeightedAverage (TimeWtAvg)	Area under the severity x time curve divided by total days of follow-up
Refractoriness	TimeNotAtGoal	Consecutive days not at goal
	Count (Ct)NotAtGoal Maximum Cluster (MaxClust)Days	Consecutive readings not at goal Longest cluster in the patient’s medical record
Tendency to relapse	Number of clusters (NumClust)	Number of relapses
Lability	Coefficient of Variation (CoeffVar)	Coefficient of variation
	Mean Value Difference (MeanValDiff)	Average of the absolute difference between all consecutive pairs
Temporal trends	NetChange	Difference between the most recent value and the first value
	Lag3Deviations(Dev)	Fractional deviation of the current value from the mean of the 3 lagging values

**Table 2:** Multivariate model for COVID-19 death using oxygen saturation (O_2_Sat) parameters.

Parameter	Coefficient	Standard Error	*p*-Value	95% Confidence Interval
O_2_SatValue1	−0.0556	0.0068	0.0000	(−0.0689, −0.0423)
O_2_SatAbnAUC	−0.0002	0.0000	0.0000	(−0.0002, −0.0002)
O_2_SatNetChange	0.0098	0.0033	0.0030	(0.0034, 0.0162)
O_2_SatTimeWtAvg	−0.2119	0.0082	0.0000	(−0.2281, −0.1958)
O_2_SatNumNotAtGoal	−0.0619	0.0089	0.0000	(−0.0793, −0.0445)
O_2_SatFUDaysNotAtGoal	0.0019	0.0008	0.0150	(0.0004, 0.0034)
O_2_SatTimeNotAtGoal	0.0015	0.0013	0.2420	(−0.0010, 0.0041)
O_2_SatCtNotAtGoal	−0.1598	0.0918	0.0820	(−0.3398, 0.0201)
O_2_SatNumClust	0.1202	0.0119	0.0000	(0.0968, 0.1435)
O_2_SatMaxClustDays	−0.0001	0.0010	0.9540	(−0.0019, 0.0018)
O_2_SatLag3Dev	3.1339	0.4382	0.0000	(2.2749, 3.9928)
O_2_SatMeanValDiff	−0.2083	0.0151	0.0000	(−0.2378, −0.1788)
O_2_SatCoeffVar	0.2091	0.0112	0.0000	(0.1871, 0.2311)
Constant	23.0275	0.5770	0.0000	(21.8966, 24.1584)

n = 299,311; AUROC = 0.643.

**Table 3:** Multivariate model for COVID-19 death using systolic blood pressure (SBP) parameters.

Parameter	Coefficient	Standard Error	*p*-Value	95% Confidence Interval
SBPValue1	−0.0039	0.0009	0.0000	(−0.0056, −0.0022)
SBPAbnAUC	0.0000	0.0000	0.0000	(0.0000, 0.0000)
SBPNetChange	−0.0060	0.0004	0.0000	(−0.0069, −0.0052)
SBPTimeWtAvg	0.0092	0.0013	0.0000	(0.0066, 0.0118)
SBPNumNotAtGoal	−0.0011	0.0002	0.0000	(−0.0014, −0.0008)
SBPFUDaysNotAtGoal	0.0004	0.0000	0.0000	(0.0004, 0.0005)
SBPTimeNotAtGoal	−0.0002	0.0001	0.0090	(−0.0003, 0.0000)
SBPCtNotAtGoal	0.0100	0.0035	0.0050	(0.0030, 0.0169)
SBPNumClust	0.0039	0.0004	0.0000	(0.0032, 0.0046)
SBPMaxClustDays	−0.0003	0.0000	0.0000	(−0.0004, −0.0003)
SBPLag3Dev	0.6649	0.0879	0.0000	(0.4925, 0.8372)
SBPMeanValDiff	−0.0152	0.0028	0.0000	(−0.0207, −0.0098)
SBPCoeffVar	0.1830	0.0035	0.0000	(0.1760, 0.1899)
Constant	−5.4780	0.1413	0.0000	(−5.7550, −5.2010)

n = 310,800; R = 0.711.

**Table 4:** Multivariate model for COVID-19 death using diastolic blood pressure (DBP) parameters.

Parameter	Coefficient	Standard Error	*p*-Value	95% Confidence Interval
DBPValue1	−0.0243	0.0015	0.0000	(−0.0273, −0.0213)
DBPAbnAUC	0.0000	0.0000	0.0010	(0.0000, 0.0000)
DBPNetChange	−0.0137	0.0007	0.0000	(−0.0151, −0.0123)
DBPTimeWtAvg	−0.0329	0.0019	0.0000	(−0.0366, −0.0292)
DBPNumNotAtGoal	−0.0042	0.0007	0.0000	(−0.0055, −0.0029)
DBPFUDaysNotAtGoal	0.0002	0.0001	0.0000	(0.0001, 0.0004)
DBPTimeNotAtGoal	−0.0002	0.0002	0.3760	(−0.0005, 0.0002)
DBPCtNotAtGoal	0.0501	0.0133	0.0000	(0.0240, 0.0761)
DBPNumClust	0.0101	0.0011	0.0000	(0.0080, 0.0122)
DBPMaxClustDays	−0.0002	0.0001	0.0010	(−0.0004, −0.0001)
DBPLag3Dev	1.5364	0.0878	0.0000	(1.3642, 1.7085)
DBPMeanValDiff	−0.1277	0.0052	0.0000	(−0.1379, −0.1175)
DBPCoeffVar	0.1646	0.0037	0.0000	(0.1574, 0.1718)
Constant	0.5617	0.1176	0.0000	(0.3312, 0.7922)

n = 310,792; AUROC = 0.705.

**Table 5:** Multivariate model for COVID-19 death using body mass index (BMI) parameters.

Parameter	Coefficient	Standard Error	*p*-Value	95% Confidence Interval
BMIValue1	−0.0547	0.0053	0.0000	(−0.0650, −0.0443)
BMIAbnAUC	0.0000	0.0000	0.0000	(0.0000, 0.0000)
BMINetChange	−0.0338	0.0028	0.0000	(−0.0394, −0.0282)
BMITimeWtAvg	−0.0017	0.0058	0.7630	(−0.0131, 0.0096)
BMINumNotAtGoal	−0.0025	0.0006	0.0000	(−0.0037, −0.0013)
BMIFUDaysNotAtGoal	0.0001	0.0000	0.0000	(0.0001, 0.0002)
BMITimeNotAtGoal	−0.0001	0.0000	0.0000	(−0.0001, 0.0000)
BMICtNotAtGoal	0.0053	0.0008	0.0000	(0.0038, 0.0068)
BMINumClust	0.0493	0.0050	0.0000	(0.0395, 0.0592)
BMIMaxClustDays	0.0000	0.0000	0.4010	(−0.0001, 0.0000)
BMILag3Dev	−0.1520	0.1522	0.3180	(−0.4503, 0.1462)
BMIMeanValDiff	−0.2505	0.0165	0.0000	(−0.2828, −0.2181)
BMICoeffVar	0.0596	0.0027	0.0000	(0.0542, 0.0650)
Constant	−1.4756	0.0728	0.0000	(−1.6184, −1.3329)

n = 296,942; AUROC = 0.650.

**Table 6:** Multivariate model for COVID-19 death using estimated glomerular filtration rate (eGFR) parameters.

Parameter	Coefficient	Standard Error	*p*-Value	95% Confidence Interval
eGFRValue1	−0.0091	0.0011	0.0000	(−0.0113, −0.0070)
eGFRAbnAUC	0.0000	0.0000	0.0000	(0.0000, 0.0000)
eGFRNetChange	0.0064	0.0006	0.0000	(0.0051, 0.0076)
eGFRTimeWtAvg	−0.0096	0.0012	0.0000	(−0.0119, −0.0072)
eGFRNumNotAtGoal	−0.0054	0.0009	0.0000	(−0.0072, −0.0036)
eGFRFUDaysNotAtGoal	0.0002	0.0000	0.0000	(0.0001, 0.0002)
eGFRTimeNotAtGoal	−0.0001	0.0000	0.0010	(−0.0001, 0.0000)
eGFRCtNotAtGoal	0.0070	0.0010	0.0000	(0.0050, 0.0090)
eGFRNumClust	0.0879	0.0037	0.0000	(0.0807, 0.0952)
eGFRMaxClustDays	0.0000	0.0000	0.7070	(−0.0001, 0.0001)
eGFRLag3Dev	−0.0054	0.0568	0.9250	(−0.1166, 0.1059)
eGFRMeanValDiff	0.0058	0.0020	0.0030	(0.0019, 0.0097)
eGFRCoeffVar	0.0198	0.0010	0.0000	(0.0178, 0.0219)
Constant	−2.0081	0.0567	0.0000	(−2.1192, −1.8970)

n = 294,314; AUROC = 0.691.

**Table 7:** Multivariate model for COVID-19 death using albuminuria (Alb) parameters.

Parameter	Coefficient	Standard Error	*p*-Value	95% Confidence Interval
AlbValue1	−1.2062	0.0428	0.0000	(−1.2902, −1.1223)
AlbAbnAUC	−0.0015	0.0002	0.0000	(−0.0019, −0.0010)
AlbNetChange	0.1897	0.0252	0.0000	(0.1403, 0.2392)
AlbTimeWtAvg	−0.1302	0.0458	0.0040	(−0.2199, −0.0405)
AlbNumNotAtGoal	−0.0179	0.0024	0.0000	(−0.0226, −0.0132)
AlbFUDaysNotAtGoal	0.0007	0.0002	0.0030	(0.0002, 0.0012)
AlbTimeNotAtGoal	0.0002	0.0002	0.1760	(−0.0001, 0.0005)
AlbCtNotAtGoal	−0.0408	0.0060	0.0000	(−0.0525, −0.0291)
AlbNumClust	0.0737	0.0086	0.0000	(0.0569, 0.0905)
AlbMaxClustDays	−0.0002	0.0003	0.3730	(−0.0008, 0.0003)
AlbLag3Dev	0.8988	0.1077	0.0000	(0.6877, 1.1100)
AlbMeanValDiff	−1.3064	0.0959	0.0000	(−1.4943, −1.1185)
AlbCoeffVar	0.0633	0.0029	0.0000	(0.0577, 0.0689)
Constant	2.3914	0.1173	0.0000	(2.1615, 2.6214)

n = 286,066; AUROC = 0.693.

**Table 8:** Multivariate model for COVID-19 death using hematocrit (HCT) parameters.

Parameter	Coefficient	Standard Error	*p*-Value	95% Confidence Interval
HCTValue1	−0.0312	0.0042	0.0000	(−0.0393, −0.0230)
HCTAbnAUC	−0.0001	0.0000	0.0000	(−0.0001, 0.0000)
HCTNetChange	−0.0437	0.0029	0.0000	(−0.0493, −0.0380)
HCTTimeWtAvg	−0.0249	0.0045	0.0000	(−0.0337, −0.0161)
HCTNumNotAtGoal	−0.0065	0.0009	0.0000	(−0.0083, −0.0046)
HCTFUDaysNotAtGoal	0.0005	0.0001	0.0000	(0.0003, 0.0008)
HCTTimeNotAtGoal	0.0002	0.0001	0.0280	(0.0000, 0.0005)
HCTCtNotAtGoal	−0.0100	0.0025	0.0000	(−0.0149, −0.0050)
HCTNumClust	0.0406	0.0049	0.0000	(0.0310, 0.0503)
HCTMaxClustDays	−0.0004	0.0002	0.0050	(−0.0007, −0.0001)
HCTLag3Dev	0.4425	0.1106	0.0000	(0.2258, 0.6592)
HCTMeanValDiff	−0.0812	0.0107	0.0000	(−0.1023, −0.0602)
HCTCoeffVar	0.0764	0.0023	0.0000	(0.0718, 0.0809)
Constant	−0.9129	0.1121	0.0000	(−1.1327, −0.6931)

n = 296,165; AUROC = 0.687.

**Table 9:** Multivariate model for COVID-19 death using low-density lipoprotein (LDL) parameters.

Parameter	Coefficient	Standard Error	*p*-Value	95% Confidence Interval
LDLValue1	0.0002	0.0006	0.8020	(−0.0011, 0.0014)
LDLAbnAUC	0.0000	0.0000	0.0000	(0.0000, 0.0000)
LDLNetChange	−0.0019	0.0003	0.0000	(−0.0025, −0.0013)
LDLTimeWtAvg	−0.0185	0.0008	0.0000	(−0.0201, −0.0168)
LDLNumNotAtGoal	0.0132	0.0028	0.0000	(0.0078, 0.0186)
LDLFUDaysNotAtGoal	−0.0001	0.0000	0.0140	(−0.0001, 0.0000)
LDLTimeNotAtGoal	−0.0001	0.0000	0.0000	(−0.0001, −0.0001)
LDLCtNotAtGoal	0.0194	0.0052	0.0000	(0.0093, 0.0295)
LDLNumClust	0.1059	0.0064	0.0000	(0.0933, 0.1185)
LDLMaxClustDays	0.0000	0.0000	0.0840	(0.0000, 0.0001)
LDLLag3Dev	−0.0997	0.0400	0.0130	(−0.1780, −0.0213)
LDLMeanValDiff	−0.0149	0.0013	0.0000	(−0.0175, −0.0123)
LDLCoeffVar	0.0203	0.0012	0.0000	(0.0180, 0.0227)
Constant	−1.5886	0.0610	0.0000	(−1.7081, −1.4691)

n = 285,423; AUROC = 0.675.

**Table 10:** Multivariate model for COVID-19 death using glycosylated hemoglobin A1c (Alc) parameters.

Parameter	Coefficient	Standard Error	*p*-Value	95% Confidence Interval
A1cValue1	−0.0044	0.0160	0.7810	(−0.0357, 0.0269)
A1cAbnAUC	0.0000	0.0000	0.0000	(0.0000, 0.0000)
A1cNetChange	−0.0168	0.0071	0.0180	(−0.0307, −0.0029)
A1cTimeWtAvg	0.0574	0.0213	0.0070	(0.0157, 0.0991)
A1cNumNotAtGoal	0.0016	0.0028	0.5600	(−0.0039, 0.0072)
A1cFUDaysNotAtGoal	0.0001	0.0000	0.0160	(0.0000, 0.0001)
A1cTimeNotAtGoal	−0.0001	0.0000	0.0000	(−0.0001, −0.0001)
A1cCtNotAtGoal	0.0038	0.0032	0.2450	(−0.0026, 0.0101)
A1cNumClust	0.1518	0.0083	0.0000	(0.1355, 0.1680)
A1cMaxClustDays	0.0002	0.0000	0.0000	(0.0001, 0.0002)
A1cLag3Dev	−0.1333	0.1004	0.1840	(−0.3300, 0.0634)
A1cMeanValDiff	−0.2625	0.0399	0.0000	(−0.3406, −0.1843)
A1cCoeffVar	0.0223	0.0024	0.0000	(0.0175, 0.0270)
Constant	−3.5018	0.0793	0.0000	(−3.6573, −3.3464)

n = 260,505; AUROC = 0.644.

**Table 11:** Multivariate model for COVID-19 death using high-density lipoprotein (HDL) parameters.

Parameter	Coefficient	Standard Error	*p*-Value	95% Confidence Interval
HDLValue1	−0.0005	0.0020	0.7910	(−0.0046, 0.0035)
HDLAbnAUC	0.0000	0.0000	0.0760	(0.0000, 0.0000)
HDLNetChange	−0.0012	0.0010	0.2210	(−0.0032, 0.0007)
HDLTimeWtAvg	0.0153	0.0023	0.0000	(0.0108, 0.0198)
HDLNumNotAtGoal	0.0189	0.0026	0.0000	(0.0137, 0.0241)
HDLFUDaysNotAtGoal	0.0000	0.0000	0.3880	(−0.0001, 0.0000)
HDLTimeNotAtGoal	0.0000	0.0000	0.1120	(−0.0001, 0.0000)
HDLCtNotAtGoal	0.0015	0.0037	0.6750	(−0.0057, 0.0088)
HDLNumClust	0.0966	0.0062	0.0000	(0.0844, 0.1088)
HDLMaxClustDays	0.0001	0.0000	0.0000	(0.0000, 0.0001)
HDLLag3Dev	−0.2636	0.0731	0.0000	(−0.4068, −0.1203)
HDLMeanValDiff	−0.0698	0.0037	0.0000	(−0.0771, −0.0625)
HDLCoeffVar	0.0427	0.0017	0.0000	(0.0393, 0.0461)
Constant	−3.9803	0.0538	0.0000	(−4.0857, −3.8748)

n = 286,325; AUROC = 0.631.

**Table 12:** Multivariate model for COVID-19 death based on baseline metabolic profiles.

Parameter	Coefficient	Standard Error	*p*-Value	95% Confidence Interval
O_2_SatValue1	−0.0731	0.0071	0.0000	(−0.0869,−0.0592)
SBPValue1	−0.0040	0.0009	0.0000	(−0.0058, −0.0023)
DBPValue1	−0.0082	0.0018	0.0000	(−0.0117, −0.0048)
BMIValue1	−0.0254	0.0017	0.0000	(−0.0287, −0.0221)
ALTValue1	−0.0036	0.0010	0.0010	(−0.0056, −0.0015)
AlbValue1	−0.5831	0.0289	0.0000	(−0.6396, −0.5265)
HCTValue1	−0.0115	0.0027	0.0000	(−0.0169, −0.0062)
A1cValue1	0.0324	0.0067	0.0000	(0.0192, 0.0456)
HDLValue1	−0.0031	0.0008	0.0000	(−0.0047, −0.0015)
O_2_SatTimeWtAvg	−0.1485	0.0092	0.0000	(−0.1664, −0.1305)
SBPTimeWtAvg	0.0297	0.0013	0.0000	(0.0271, 0.0323)
DBPTimeWtAvg	−0.0424	0.0021	0.0000	(−0.0466, −0.0383)
eGFRTimeWtAvg	−0.0131	0.0009	0.0000	(−0.0148, −0.0115)
ALTTimeWtAvg	−0.0186	0.0021	0.0000	(−0.0228, −0.0145)
LDLTimeWtAvg	−0.0074	0.0006	0.0000	(−0.0086, −0.0062)
O_2_SatAbnAUC	−0.0001	0.0000	0.0000	(−0.0001, −0.0001)
eGFRAbnAUC	0.0000	0.0000	0.0000	(0.0000, 0.0000)
LDLAbnAUC	0.0000	0.0000	0.0000	(0.0000, 0.0000)
ALTAbnAUC	0.0000	0.0000	0.0000	(0.0000, 0.0000)
SBPNumNotAtGoal	−0.0010	0.0002	0.0000	(−0.0014, −0.0006)
BMINumNotAtGoal	−0.0018	0.0004	0.0000	(−0.0025, −0.0011)
LDLNumNotAtGoal	0.0075	0.0021	0.0000	(0.0033, 0.0116)
HDLFUDaysNotAtGoal	0.0000	0.0000	0.0000	(0.0000, 0.0000)
AlbCtNotAtGoal	−0.0128	0.0043	0.0030	(−0.0213, −0.0043)
LDLTimeNotAtGoal	0.0000	0.0000	0.0460	(0.0000, 0.0000)
HDLTimeNotAtGoal	0.0000	0.0000	0.0010	(0.0000, 0.0000)
eGFRMeanValDiff	0.0126	0.0026	0.0000	(0.0075, 0.0176)
AlbMeanValDiff	−0.4072	0.1128	0.0000	(−0.6282, −0.1862)
SBPCoeffVar	0.0643	0.0036	0.0000	(0.0572, 0.0715)
BMICoeffVar	0.0152	0.0024	0.0000	(0.0104, 0.0199)
AlbCoeffVar	0.0136	0.0030	0.0000	(0.0077, 0.0194)
HCTCoeffVar	0.0137	0.0023	0.0000	(0.0092, 0.0181)
SBPNumClust	0.0021	0.0004	0.0000	(0.0013, 0.0029)
eGFRNumClust	0.0112	0.0033	0.0010	(0.0047, 0.0177)
ALTNumClust	0.0254	0.0059	0.0000	(0.0140, 0.0369)
AlbNumClust	−0.0227	0.0074	0.0020	(−0.0372, −0.0082)
HCTNumClust	−0.0150	0.0040	0.0000	(−0.0228, −0.0072)
LDLNumClust	0.0304	0.0064	0.0000	(0.0179, 0.0429)
A1cNumClust	0.0423	0.0067	0.0000	(0.0292, 0.0554)
O_2_SatLag3Dev	3.8382	0.4806	0.0000	(2.8962, 4.7803)
SBPLag3Dev	0.3222	0.1195	0.0070	(0.0879, 0.5565)
DBPLag3Dev	0.3167	0.1249	0.0110	(0.0718, 0.5616)
eGFRLag3Dev	−0.1797	0.0531	0.0010	(−0.2839, −0.0756)
LDLLag3Dev	0.1140	0.0317	0.0000	(0.0519, 0.1761)
O_2_SatNetChange	0.0098	0.0039	0.0120	(0.0022, 0.0175)
AlbNetChange	0.1491	0.0234	0.0000	(0.1032, 0.1949)
HCTNetChange	−0.0161	0.0025	0.0000	(−0.0210, −0.0112)
LDLNetChange	−0.0006	0.0002	0.0180	(−0.0011, −0.0001)
A1cNetChange	0.0260	0.0063	0.0000	(0.0136, 0.0384)
Constant	23.4159	0.7186	0.0000	(22.0075, 24.8244)

n = 239,393; AUROC = 0.785.

## Data Availability

Data cannot be shared publicly because it involves sensitive human subject information. Data may be available for researchers who meet the criteria for access to confidential data after evaluation from the affiliated IRB and VA Research and Development Committees. As a national VA legal policy (VHA Directive 2605.01), VA will only share patient data if there is a fully executed contractual agreement in place for the specific project. A common contractual mechanism utilized for this type of sharing is a Cooperative Research and Development Agreement (CRADA). These contracts are typically negotiated in collaboration with the national VA Office of General Council (OGC) and attorneys from the collaborating institution. These national sharing policies and standards also apply to de-identified data. In addition, if a contract is in place allowing the sharing of de-identified data outside of VA, then VA national policy (Directive 1605.01) states that de-identification certification needs to be met by Expert Determination. The expert determination requires independent assessment from an experienced master’s or PhD in biostatistics, from a third party not involved in the project, and may require outside funding to support. In addition, for an outside entity to perform research on VA patient data, IRB as well as VA Research and Development Committee approval is required for the specific project. Data requests may be sent to VA Information Resource Center (VIReC) Building 18 Hines VA Hospital (151V) 5000 S. 5th Avenue Hines, IL 60141-3030 708-202-2415 (fax) virec@va.gov.

## Abbreviations

The following abbreviations are used in this manuscript:

A1c	glycosylated hemoglobin
ALB	albuminuria
ALT	alanine aminotransferase
AUC	area under the severity x time curve
AUROC	area under the receiver operating curve
BMI	body mass index
BP	blood pressure
CDW	Corporate Data Warehouse
Charl2Yrs	2-year Charlson Comorbidity Index score
CharlEver	lifetime Charlson Comorbidity Index score
CMs	clinical measurements
COVID-19	coronavirus disease 2019
CSDR	VA’s COVID-19 Shared Data Resource
DBP	diastolic blood pressure
eGFR	estimated glomerular filtration rate
Elix2Yrs	Elixhauser 2-year score
ElixEver	lifetime Elixhauser score
HCT	hematocrit
HDL	high-density lipoprotein
LDL	low-density lipoprotein
MAFLD	metabolic-dysfunction-associated fatty liver disease
O_2_Sat	oxygen saturation
PDeathLabs	predicted probability of death
SBP	systolic blood pressure
TRIPOD	transparent reporting of a multivariate prediction model for individual prognosis or diagnosis
U.S.	United States
